# Case Report: Successful treatment of triple primary cancers (lung, stomach, and intestine) using sintilimab combined with chemotherapy and targeted therapy

**DOI:** 10.3389/fimmu.2025.1614911

**Published:** 2025-08-05

**Authors:** Qi-Qing Zhang, Xin-Xin Luo, Hua Chen, Wei Wang, Yi-Fan Xie, Hong-Li Qiao, Hao-Jun Miao, Zheng-Quan Feng

**Affiliations:** ^1^ Department of Oncology, Tongde Hospital of Zhejiang Province, Hangzhou, Zhejiang, China; ^2^ College of Integrated Traditional Chinese and Western Medicine Clinical Medicine, Zhejiang Chinese Medical University, Hangzhou, Zhejiang, China; ^3^ Department of Pathology, Tongde Hospital of Zhejiang Province, Hangzhou, China

**Keywords:** chemotherapy, immunotherapy, multiple primary cancers, sintilimab, targeted therapy

## Abstract

This case report presents a rare instance of synchronous multiple primary cancers involving lung adenocarcinoma with bone metastasis, gastric signet-ring cell carcinoma, and rectal cancer. The 64-year-old male patient was treated with a combination of sintilimab, chemotherapy, and targeted therapy. Following a multidisciplinary team consultation, systemic treatment with sintilimab, oxaliplatin, and capecitabine was initiated concurrently while furmonertinib targeted therapy continued. After six cycles, the lung lesions showed significant reduction, while the gastric and intestinal tumors remained stable. The patient transitioned to maintenance therapy and achieved sustained disease control without severe adverse effects. This case highlights the potential of an integrated immunotherapy and chemotherapy approach for treating multiple primary malignancies with distinct pathological origins. report also underscores the need for individualized, biomarker-driven treatment strategies and further research on optimal therapeutic combinations for synchronous multiple primary cancers.

## 1 Introduction

Multiple primary cancers—also known as compound or repeated cancers—refer to the occurrence of two or more independent primary cancers in the same individual, either simultaneously or sequentially. Based on the time interval between occurrences, they are classified as metachronous or synchronous multiple primary cancers (1). Multiple primary cancers are defined according to guidelines from the Surveillance Epidemiology and End Results (SEER) Program and the International Association of Cancer Registries (IACR) along with the International Agency for Research on Cancer (IARC) (2–4). The incidence of multiple primary cancers in the cancer population ranges from 2.4–8%, and can reach as high as 17% after 20 years of follow-up (5). Currently, no standardized treatment strategy exists for multiple primary cancers, and research on this condition remains less extensive than that on single primary cancers. Diagnosing and treating multiple primary cancers require a comprehensive assessment of factors such as the clinical stage and pathological type of each lesion; radical treatment is prioritized, particularly for localized tumors (5). When surgical resection is not feasible, comprehensive treatment options—including chemotherapy, radiotherapy, immunotherapy, and targeted therapy—should be considered. Although simultaneous surgical treatment for multiple primary cancers in the digestive system has been documented (6), reports of non-surgical treatment using the same regimen for synchronous multiple primary cancers of different pathological types across multiple systems are rare.

This article presents a successfully managed case of synchronous multiple primary cancers, including lung adenocarcinoma with bone metastasis, gastric signet-ring cell carcinoma, and rectal cancer, treated with a uniform regimen. The case’s characteristics, diagnostic process, and therapeutic approach are described to enhance the understanding and management of multiple primary cancers.

## 2 Case summary

In April 2024, the patient presented to our hospital with progressive left hip pain accompanied by numbness in the left lower limb. An external hospital Emission Computed Tomography scan (ECT) indicated abnormal bone salt metabolism in the right second anterior rib, left iliac bone, left acetabulum, and right sacroiliac joint, suggestive of possible metastasis.

**Figure 1:**
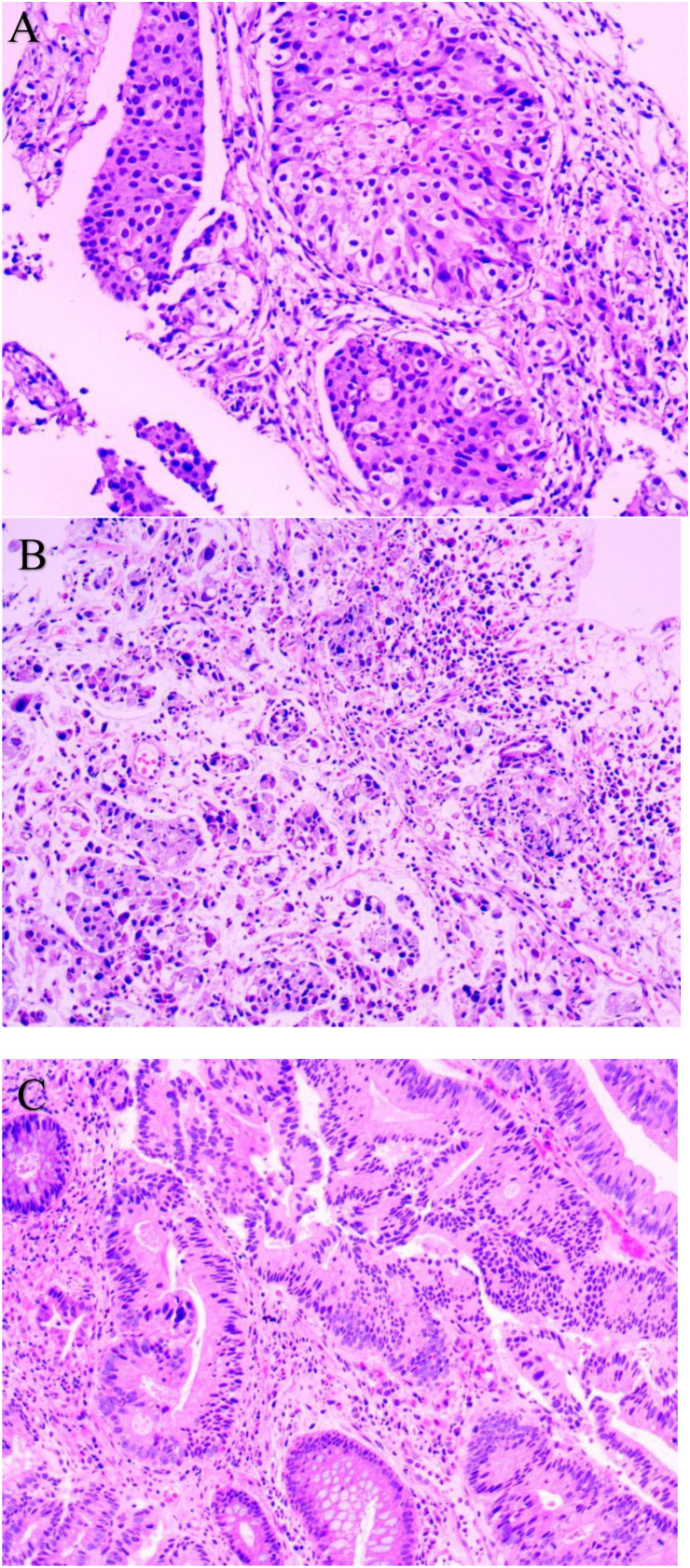
Pathological Examination (HE staining): **(A)** Lung Biopsy (200×); **(B)** Gastric Biopsy (200×); **(C)** Rectal Biopsy (200×).

Approximately four years ago (April 2021), the patient presented to an external hospital with a one-week history of cough. Contrast-enhanced chest CT revealed a left lung mass. On May 20, 2021, a left lung biopsy confirmed poorly differentiated non-small cell lung cancer (NSCLC) consistent with adenocarcinoma (cT4N3M0, Stage IIIC). Immunohistochemistry (IHC) showed CK7(+), TTF-1(+), NapsinA(+), P40(-), and CK5/6(-) (**Figure 1A**). Molecular testing identified EGFR exon 18 (G719X) and exon 20 (S768I) mutations. Oral afatinib 40 mg once daily (QD) was initiated on June 1, 2021. One month later, restaging demonstrated partial response (PR). Due to grade 1 rash (affecting face and lower extremities) and abdominal pain, the dose was reduced to 30 mg QD. After five months of afatinib, progressive disease in the left lung prompted local pulmonary radiotherapy (November 24, 2021 - January 5, 2022). Restaging four months post-radiotherapy confirmed stable disease (SD).Seven months post-radiotherapy, recurrent left lung progression occurred. Bronchoscopic biopsy at an external institution revealed a small cancer cluster in the left superior lingular bronchus (consistent with adenocarcinoma; specimen insufficient for genetic testing). Due to intolerable afatinib-related toxicity, therapy was switched to oral furmonertinib 80 mg QD in October 2022, which remains ongoing. The patient has a >40 pack-year smoking history. Family history is negative for hereditary cancer syndromes including familial adenomatous polyposis and Lynch syndrome, etc.

Physical examination showed tenderness in the left hip, accompanied by numbness in the left lower limb. Neurological examination showed negative pathological signs, and cardiopulmonary auscultation was unremarkable, and no palpable superficial lymphadenopathy was detected.

After admission, laboratory test results showed elevated tumor markers and severe anemia. Pre-treatment laboratory test results were as follows: carcinoembryonic antigen, 5.13 ng/mL; carbohydrate antigen 125, 33.16 U/mL; carbohydrate antigen 19-9, 19.45 IU/mL; cytokeratin 19 fragment, 21.42 ng/mL; neuron-specific enolase, 31.5 ng/mL; white blood cell count, 7.6 × 10^9^/L; neutrophil count, 5.3 × 10^9^/L; red blood cell count, 2.09 × 10¹²/L; hemoglobin, 52 g/L; fecal occult blood, OB++; urine bacteria, ++; and D-dimer, 7.33 mg/L.

**Figure 2:**
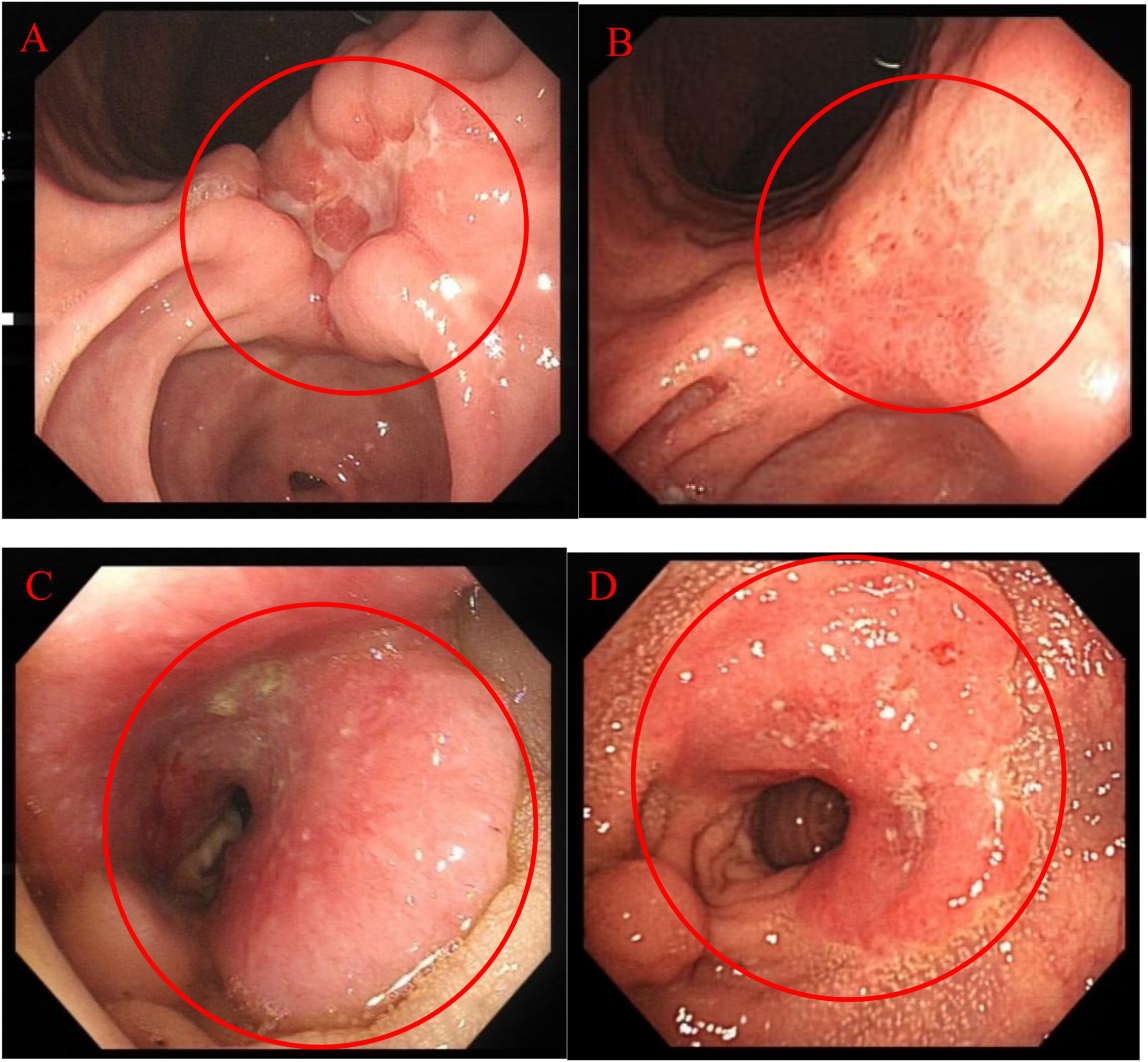
Endoscopic Examination: **(A)** 2024.04.29 Gastroscopy; **(B)** 2024.09.13 Gastroscopy; **(C)** 2024.04.29 Colonoscopy; **(D)** 2024.09.13 Colonoscopy.

At the same time, on April 29, 2024, gastroscopy revealed an ulcer approximately 3 cm in diameter was observed near the posterior wall, with irregular surrounding elevation and a hard biopsy texture (**Figure 2A**). Gastric biopsy showed that (Gastric angle) poorly cohesive carcinoma with a minor component of signet-ring cell carcinoma. Helicobacter pylori (HP): (-) (**Figure 1B**). IHC: CK7 (-), TTF-1 (-), NapsinA (-), P53 (mutant type, loss of expression), Ki-67 (+, 70%), Muc-2 (+), Muc-6 (+, focal), Muc-5AC (+, partial), Villin (+), SATB2 (+). Fluorescence *in situ* hybridization: Her-2 negative. Microsatellite stability (MSS). PD-L1 IHC: TPS 10%, CPS 15. While colonoscopy revealed a rectal mass approximately 7 cm from the anus encircling 5/6 of the intestinal lumen, causing stenosis was observed. The endoscope could barely pass through, and the surface appeared congested with a fragile biopsy texture (**Figure 2C**). High-grade intraepithelial neoplasia of the glandular epithelium with focal carcinoma, consistent with a primary rectal tumor based on clinical and IHC findings. IHC: CerbB-2 (1+), CDX-2 (+), MSH2 (+), MSH6 (+), MLH1 (+), PMS2 (+), P53 (+, 70%), Ki-67 (+, 80% in hotspot areas), CK7 (-), CK20 (+), TTF-1 (-), NapsinA (-), SATB2 (+) (**Figure 1C**).

Metastatic cancer usually retains the pathological type, immunohistochemical spectrum, molecular features, etc. of the primary lesion, while lung cancer, gastric cancer, and rectal cancer lesions in this patient are independent of each other. Based on the above medical history and laboratory tests, the patient was diagnosed with non-small cell lung adenocarcinoma with bone metastasis, gastric signet-ring cell carcinoma, and rectal cancer. Additionally, the patient presented with severe anemia.

Following multidisciplinary team consultation, the patient continued oral targeted therapy with Furmonertinib. Due to the patient’s anemia and other conditions, he was unable to tolerate bone radiotherapy. Therefore, incadronate 10mg every 4 weeks were given to prevent bone metastasis. Regarding stomach cancer and rectal cancer, after contraindications were ruled out, systemic treatment was initiated on May 22, 2024, consisting of: Oxaliplatin 150 mg on day 1, Capecitabine 1.5 g twice daily on days 1–14, Sintilimab 0.2 g on Day 1, and Treatment cycle: Every 3 weeks. After 2 cycles, the lung, gastric, and intestinal lesions showed reduction in size, while anemia and pain improved. After 4 cycles, the lung lesions further decreased, while the gastric and intestinal lesions remained stable. After 6 cycles, re-evaluation indicated stable disease (SD). The patient was transitioned to maintenance therapy with: Capecitabine 1.5 g twice daily on days 1–14 and Sintilimab 0.2 g on Day 1 every 3 weeks. After 3 months, re-evaluation confirmed SD, and the patient remains under surveillance with tumor assessments indicating ongoing SD.

**Figure 3:**
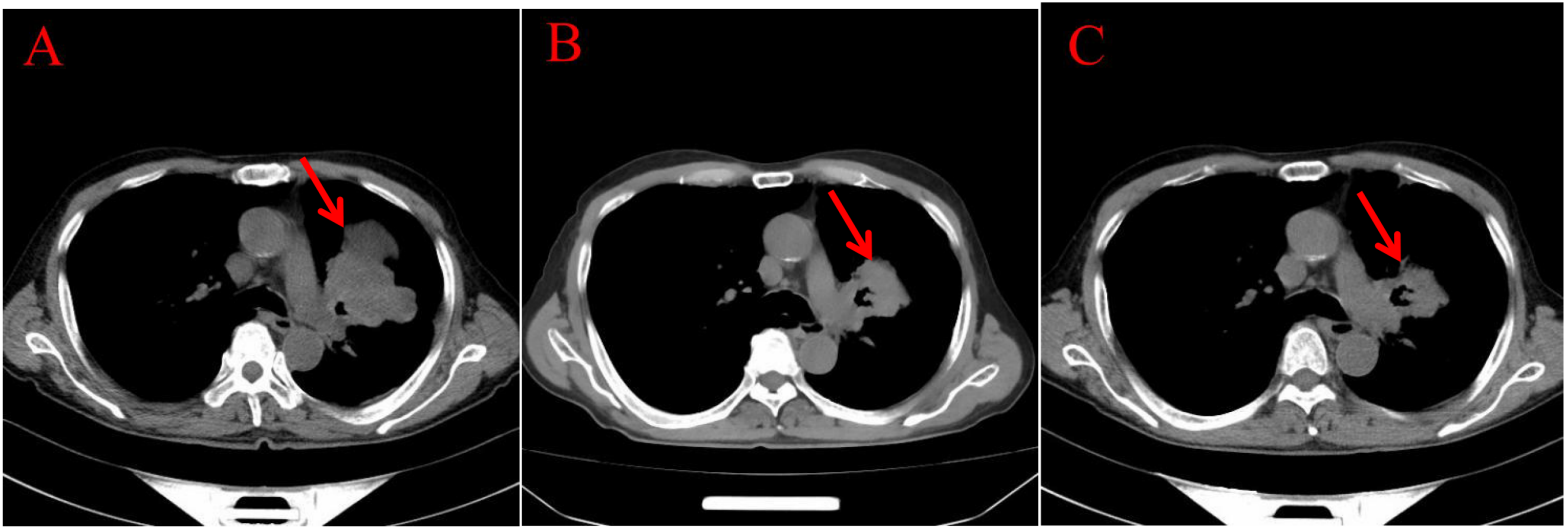
Imaging examinations of the changes in lung lesions: **(A)** (2024.5.2) Central type lung cancer with obstructive pneumonia and atelectasis in the upper lobe of the left lung, similar to the mass in the previous film (January 29, 2024). A huge soft tissue density shadow with a size of approximately 80 * 54mm can be seen near the hilum of the upper lobe of the left lung. **(B)** (2024.12.11) Central type lung cancer with obstructive pneumonia and atelectasis in the upper lobe of the left lung, with a reduced mass compared to the previous film (May 2, 2024). A huge soft tissue density shadow with a size of approximately 52 * 49mm can be seen near the hilum of the upper lobe of the left lung. **(C)** (2024.3.25) Central type lung cancer with obstructive pneumonia and atelectasis in the upper lobe of the left lung, with a slightly smaller mass compared to the previous film (December 11, 2024). There is a patchy shadow around the local bronchus in the upper lobe of the left lung, with unclear boundaries.

**Figure 4:**
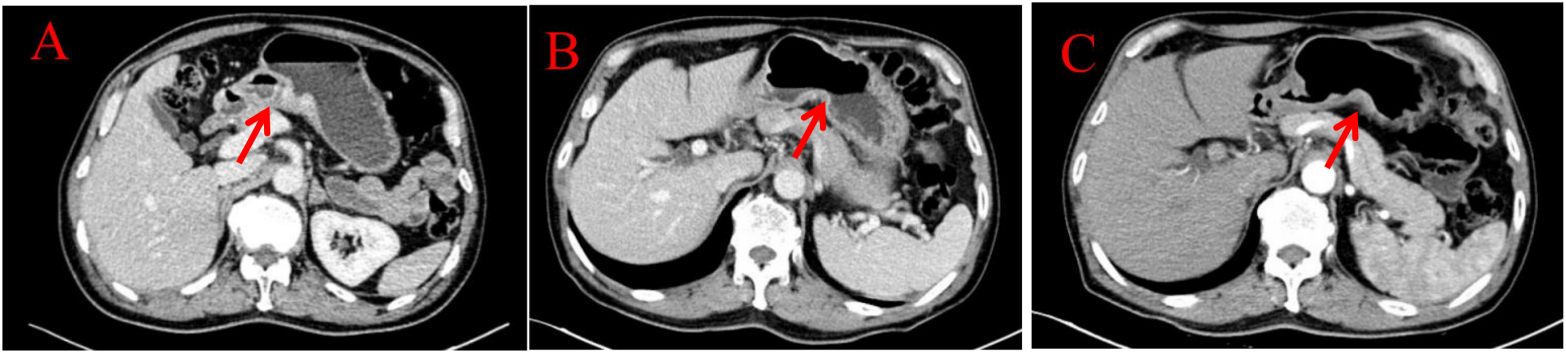
Imaging examinations of the changes in gastric lesions: **(A)** (2024.4.18) The gastric cavity is poorly filled, and there is a suspected slight thickening of the gastric wall locally. **(B)** (2024.12.10) The gastric cavity is poorly filled, and the gastric wall is thickened, similar to before. **(C)** (2025.3.26) The gastric cavity is poorly filled, and the gastric wall at the lesser curvature of the stomach is slightly thickened, similar to before.

**Figure 5:**
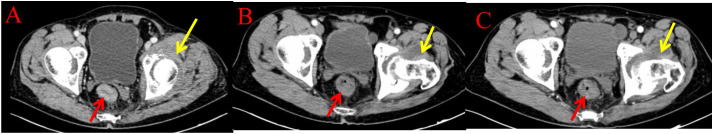
Imaging examinations of the changes in intestinal and bone lesions: **(A)** (2024.4.18) The rectal wall is locally thickened, and the enhanced examination shows mild enhancement, with a thickness of about 15mm and a cumulative length of about 46mm. The outer wall is slightly rough. Left acetabular bone resorption and destruction, accompanied by the formation of a soft tissue mass shadow. **(B)** (2024.12.10) The rectal wall is locally thickened, and the enhanced examination shows mild enhancement. The intestinal lumen is narrowed, and the outer wall is slightly rough. Left acetabular bone resorption and destruction, accompanied by the formation of a soft tissue mass shadow. **(C)** (2025.3.26) The rectal wall is locally thickened, and the enhanced examination shows mild enhancement. The intestinal lumen is narrowed, and the outer wall is slightly rough. Left acetabular bone resorption and destruction, accompanied by the formation of a soft tissue mass shadow.

**Figure 6:**
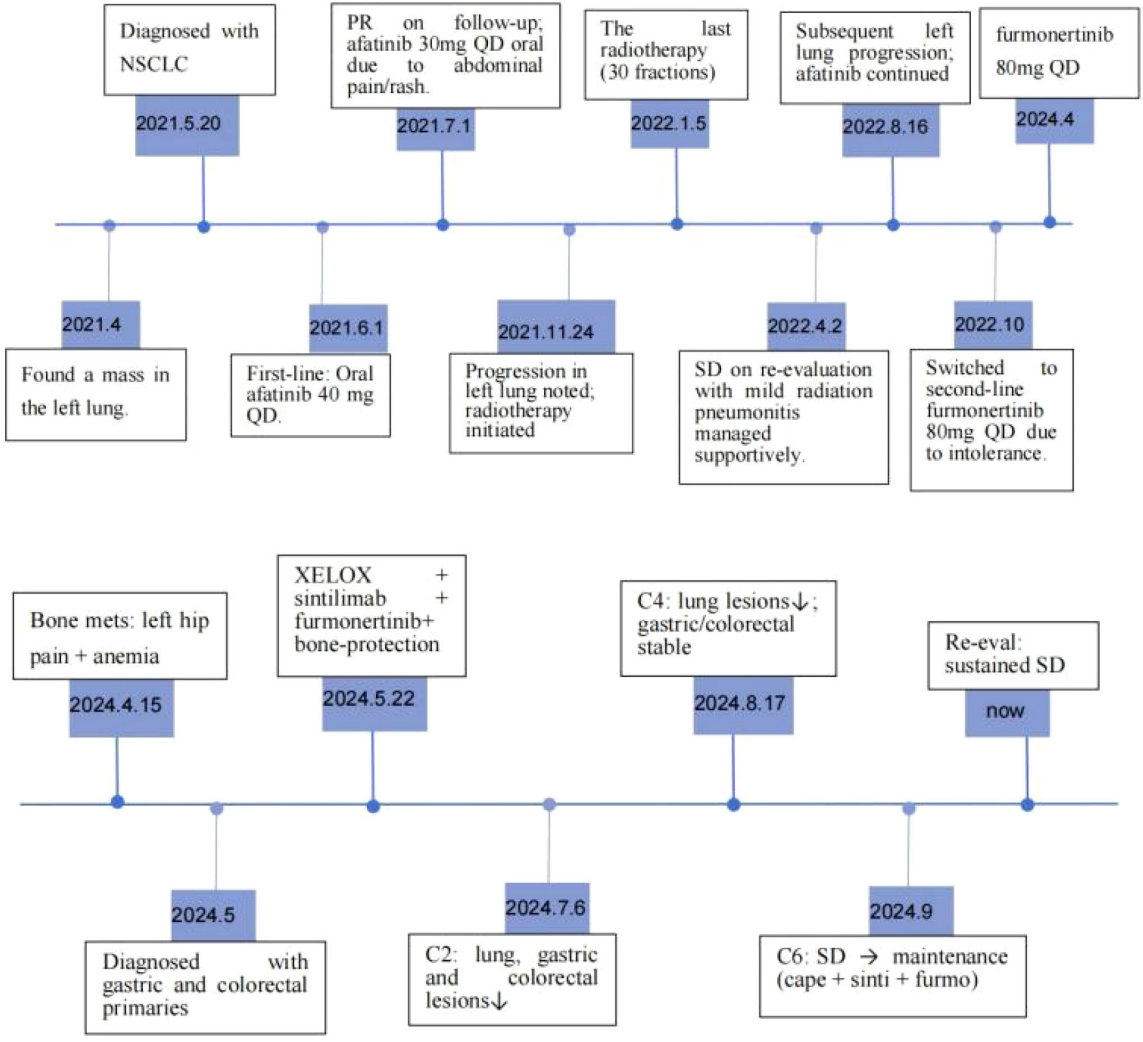
The timeline of the diagnosis and treatment process.

Endoscopic Examination is shown in **Figure 2**. Pathological Examination is shown in **Figure 1**. Imaging examinations of the changes in lung, gastric, intestinal and bone lesions are shown in **Figures 3**–**5**. The timeline of the diagnosis and treatment process is shown in **Figure 6**.

## 3 Discussion

### 3.1 Precise diagnosis of triple primary cancers (lung, stomach, and colorectum): multi-dimensional differentiation based on pathology, chronology, and IHC

The diagnostic complexity in this case lay in determining whether the lung, gastric, and colorectal cancers represented primary lesions or metastases. We concluded that all three lesions were primary based on three key dimensions: pathological morphology differences, temporal intervals with anatomical independence, and immunohistochemical profiles. The rationale is as follows:

1. Pathological morphological differences. The lung cancer lesion is poorly differentiated lung adenocarcinoma without signet ring cell description. Gastric cancer lesions with low adhesion and signet ring cell carcinoma are a special type of gastric cancer; The rectal cancer lesion is a high-grade adenocarcinoma of the glandular epithelium, which is a typical development pattern of colorectal cancer(CRC).
2. Time interval and site independence. Diagnosed with left lung adenocarcinoma in 2021. Diagnosed with gastric angle low adhesion cancer in 2024, containing signet ring cell components, with a 3-year interval from lung cancer. Diagnosed with rectal adenocarcinoma in 2024, discovered at the same time as gastric cancer but with independent pathological features.
3. The immunohistochemical lineage completely deviates from the transfer pattern. Lung cancer markers CK7 (+), TTF-1 (+), and NapsinA (+) are typical lung adenocarcinoma phenotypes, while P40 (-) and CK5/6 (-) can exclude squamous cell carcinoma. The gastric cancer markers CK7 (-), TTF-1 (-), and NapsinA (-) completely exclude lung derived metastasis, while Muc-2 (+), Muc-5AC (+), and Muc-6 (+) are gastric mucinous phenotypes, indicating gastric primordia. Although Villin (+) and SATB2 (+) suggest a tendency towards intestinal differentiation, P53 in gastric cancer is (mutant, absent expression), while in colorectal cancer P53 (+, 70%), P53 expression is inconsistent, still supporting gastric primordia. The rectal cancer markers CK20 (+), CDX-2 (+), and SATB2 (+) are classic colorectal adenocarcinoma phenotypes, while CK7 (-), TTF-1 (-), and NapsinA (-) can exclude lung metastasis.

### 3.2 Precision and standardized management of triple primary cancers (lung, gastric, and colorectal)

The treatment of multiple primary cancers follows principles similar to those for single primary cancers but differs significantly from the management of metastatic or recurrent cancers. Treatment decisions are influenced by pathological type, organ involvement, and timing of tumor onset (synchronous vs. metachronous); accurate and timely diagnosis is therefore essential. Currently, histopathological examination remains the routine approach for diagnosing and differentiating multiple primary cancers. This patient was diagnosed with lung adenocarcinoma with bone metastasis, gastric signet-ring cell carcinoma, and primary rectal cancer, each exhibiting distinct pathological features. The lung adenocarcinoma and dual primary gastrointestinal malignancies were detected independently at different times. The presence of metastasis further complicated treatment planning. Cases involving multiple primary malignancies with distinct pathological types are rare, and documented successful treatment outcomes remain scarce.

Due to the fact that lung cancer treatment was already a second-line option when the patient arrived at our hospital, and the discovery of dual primary cancers of the gastrointestinal tract with bone metastasis, diagnosis and treatment were difficult.

#### 3.2.1 Treatment of lung adenocarcinoma

At initial diagnosis, the patient presented with Stage IIIC (T4N3M0) unresectable NSCLC. Genetic testing identified G719X and S768I. Afatinib was selected as first-line therapy based on the 2021 Chinese Society of Clinical Oncology (CSCO) NSCLC Guidelines (7) and the patient’s preferences. Afatinib, a second-generation irreversible EGFR tyrosine kinase inhibitor (TKI), targets EGFR mutation-positive NSCLC by broadly inhibiting receptors including EGFR, HER2, and HER4. These uncommon EGFR mutations (G719X/S768I) typically exhibit reduced sensitivity to first-generation EGFR-TKIs (gefitinib, erlotinib). Substantial clinical and real-world evidence supports afatinib’s efficacy against uncommon mutations (e.g., G719X, S768I, L861Q) in advanced NSCLC. Consequently, afatinib was initiated on June 1, 2021, after the patient declined chemotherapy, radiotherapy, and immunotherapy. Dose reduction was implemented after one month due to adverse events (abdominal pain, rash). Five months later, left lung progression occurred. Per CSCO guidelines, afatinib was continued alongside local radiotherapy (30 fractions administered from November 24, 2021, to January 5, 2022). Four months post-radiotherapy, disease was stable with mild radiation pneumonitis.

Seven months post-radiotherapy, the left lung tumor progressed again. A repeat biopsy per CSCO guidelines yielded insufficient tissue for genetic testing, so afatinib was continued. Due to intolerable afatinib-related toxicity, therapy was switched to furmonertinib in October 2022. Furmonertinib, a third-generation EGFR-TKI, selectively targets EGFR sensitizing mutations and T790M resistance mutations. It is indicated for locally advanced or metastatic NSCLC and demonstrates favorable blood-brain barrier penetration. Despite lacking confirmed T790M mutation, furmonertinib was initiated as second-line therapy after thorough patient discussion and informed consent.

Following 18 months of second-line furmonertinib, the patient developed left hip pain. Presentation to our institution on April 15, 2024, led to a diagnosis of bone metastasis, most likely originating from the lung cancer. The 2024 CSCO NSCLC Guidelines (8) recommend a tripartite approach for bone metastasis: radiotherapy, chemotherapy, and bone-protective therapy. Due to concurrent anemia, radiotherapy was deferred. Incadronate disodium was initiated for bone metastasis control. Given stable disease in the left lung and absence of significant adverse events, furmonertinib was continued.

#### 3.2.2 Treatment of gastric and rectal cancer

Given the patient’s advanced disease status with lung cancer bone metastasis and synchronous gastric and colorectal primary carcinomas, radical surgical resection was deemed non-curative, offering limited benefit with unquantifiable risks. Systemic therapy was prioritized. Per the 2024 CSCO Gastric Cancer Guidelines (9), first-line treatment for metastatic HER2-negative gastric cancer with PD-L1 CPS ≥5 recommends XELOX (capecitabine + oxaliplatin) combined with sintilimab. Concurrently, the 2024 CSCO rectal Cancer Guidelines (10) (palliative care group) recommend first-line regimens FOLFOX (oxaliplatin + leucovorin + fluorouracil), CAPEOX (capecitabine + oxaliplatin), or FOLFIRI (irinotecan + leucovorin + fluorouracil) ± bevacizumab. Consequently, an oxaliplatin plus capecitabine chemotherapy backbone aligned with guideline recommendations for first-line palliative treatment of unresectable gastric and rectal cancers. Bevacizumab, a recombinant humanized monoclonal antibody, inhibits tumor angiogenesis by binding vascular endothelial growth factor (VEGF). Sintilimab, a fully human IgG4 monoclonal antibody targeting PD-1, disrupts immune suppression by blocking PD-1/PD-L1/PD-L2 interactions, thereby activating T-cell function and enhancing antitumor immunity (11). Bevacizumab was contraindicated due to a gastric ulcer (3 cm diameter) at the gastric angle near the posterior wall observed on endoscopy, posing a risk of gastrointestinal perforation. It is indicated in combination with fluoropyrimidine and platinum-based chemotherapy for unresectable locally advanced, recurrent, or metastatic gastric or gastroesophageal junction adenocarcinoma. Therefore, on May 22, 2024, first-line therapy with sintilimab combined with oxaliplatin and capecitabine was initiated.

Following six cycles of this combined regimen, significant regression was observed not only in the gastric and colorectal lesions but also in the pulmonary lesions, with no significant treatment-related toxicity, demonstrating the efficacy of sintilimab against lung, gastric, and colorectal malignancies. The patient subsequently transitioned to maintenance therapy with capecitabine plus sintilimab. Current disease assessment indicates SD.

### 3.3 The era of precision oncology: the indispensable role of tumor biomarkers and molecular pathology testing

The implementation of biomarker testing, particularly the evaluation of HER2 status, programmed death-ligand 1 (PD-L1) expression, and microsatellite instability status, has significantly influenced clinical practice and patient care. In this case, negative HER2 status and positive PD-L1 expression guided the selection of a triple therapy approach combining immunotherapy, chemotherapy, and targeted therapy. Multiple studies have shown that increased expression of PD-L1 can be observed in cancers such as NSCLC, gastric cancer, and CRC (12–15). This may be related to the dysregulation of pathways such as PI3K-AKT (16) and Ras/Raf/MEK/ERK (17), which can increase the survival and proliferation of various cancer cells. Antibodies targeting PD-L1 can control immune escape and enhance adaptive immune responses, thereby killing tumor cells (18).

### 3.4 The surprising efficacy and manageable safety of sintilimab combination therapy in this case

Advances in identifying oncogenic mutations have driven the development of targeted therapies. Treatment strategies for advanced lung adenocarcinoma are typically tailored based on the presence or absence of driver gene mutations (19). Patients without driver gene mutations may receive immunotherapy with chemotherapy, anti-angiogenic therapy with chemotherapy, or platinum-based doublet chemotherapy, depending on their performance status. In contrast, patients with driver gene mutations are usually treated with targeted therapy, anti-angiogenic therapy, or a combination of both. In clinical practice, the combination of immunotherapy with EGFR-TKI is rarely employed. First, immunotherapy has demonstrated limited efficacy in patients with EGFR-mutant NSCLC. While PD-1 and PD-L1 inhibitors improve NSCLC outcomes compared to second-line chemotherapy; this benefit does not extend to the EGFR-mutant subgroup. Second, the KEYNOTE-021 (20) trial showed that while the combination of Pembrolizumab and Erlotinib was tolerable in 19 patients, the combination of Pembrolizumab and Gefitinib led to intolerable adverse reactions, with 5 of 7 patients experiencing grade 3–4 hepatotoxicity. These severe adverse reactions have raised concerns regarding the safety of combining immunotherapy with EGFR-TKIs. Crucially, in this case, however, Sintilimab combined with Furmonertinib resulted in no adverse reactions while also reducing lung lesions, which showed significant improvement compared to targeted therapy with Furmonertinib alone.

Sintilimab is a recombinant, fully human IgG4 anti–PD-1 monoclonal antibody, administered intravenously (21). The combination of Sintilimab and targeted therapy played a pivotal role in controlling lung cancer in this case. In this case, the combination of sintilimab and furmonertinib yielded surprisingly favorable outcomes in controlling the lung cancer. Notably, none of the anticipated immune-related adverse events (e.g., immune-mediated rash, hepatotoxicity, nephrotoxicity, pneumonitis, or colitis) occurred. Instead, only neutropenia (grade 1 bone marrow suppression) was observed. Furthermore, significant regression of pulmonary lesions was documented, demonstrating marked improvement compared to furmonertinib monotherapy.

Sintilimab has also demonstrated significant efficacy in treating gastric cancer. The ORIENT-16 study (22) revealed that, compared to chemotherapy alone, Sintilimab combined with chemotherapy significantly prolonged overall survival, achieving a median survival of 19 months (CPS ≥5). This represents a 50% improvement over traditional chemotherapy and a 41% reduction in mortality risk, with manageable safety. As a result, the 2023 CSCO Gastric Cancer Guidelines (23) recommended Sintilimab as a first-line treatment for all patient populations. Multiple phase III trials (24–26) have confirmed its efficacy In this case, after five cycles of treatment, follow-up gastroscopy revealed that the 3 cm ulcer in the gastric angle had healed into a scar (**Figure 2B**). Repeat biopsies showed no evidence of malignancy, achieving a pathological response, while hemoglobin reached 134g/L. Verified effective treatment of gastric cancer and improvement of anemia.

In CRC, Sintilimab’s efficacy is primarily observed in PD-1-positive patients. Thus, PD-L1 positivity may help identify those most likely to benefit from combination therapy. Immune checkpoint inhibitors targeting PD-1 have proven highly effective, leading to clinical complete response rates of up to 100% (27) in patients with CRC with deficient mismatch repair or high microsatellite instability. However, in metastatic CRC, these agents are largely ineffective as monotherapy for most patients (approximately 85%) with proficient mismatch repair or MSS CRC. Findings from the NCT04304209 study (28) indicated that adding Sintilimab to neoadjuvant chemoradiotherapy significantly increased the complete response rate in locally advanced CRC with proficient mismatch repair, with manageable safety. In this case, after five cycles of treatment, follow-up colonoscopy showed lesion reduction, achieving pathological complete response (**Figure 2D**).

The strengths of this case report include favorable treatment outcomes, the absence of significant toxic side effects, and well-reasoned treatment decisions. These findings provide valuable insights for selecting treatment strategies for patients with multiple primary cancers involving distinct pathological types across multiple organ systems. The primary limitation of this report is limited generalizability of the findings.

## 4 Conclusion

For patients with multiple primary cancers, surgical resection of some lesions remains the preferred option when surgery is indicated. However, for patients who are ineligible for surgery, or where surgery is not a viable option, identifying an effective treatment regimen to address multiple primary cancers simultaneously is crucial. In this case, a combination of chemotherapy, immunotherapy, and targeted therapy demonstrated excellent efficacy, suggesting a potential approach for the diagnosis and treatment of multiple primary cancers.

## Data Availability

The original contributions presented in the study are included in the article/supplementary files, further inquiries can be directed to the corresponding author/s.
